# Proteomic analysis of human periodontal ligament cells under hypoxia

**DOI:** 10.1186/s12953-019-0151-2

**Published:** 2019-08-31

**Authors:** Qiwen Li, Tao Luo, Wenxin Lu, Xiaoxiao Yi, Zhihe Zhao, Jun Liu

**Affiliations:** 1 State Key Laboratory of Oral Diseases & National Clinical Research Center for Oral Diseases, West China Hospital of Stomatology, Sichuan University, No.14, 3rd Section, South Renmin Road, Chengdu, 610041 China; 2 Sichuan Cancer Hospital & Institute, Sichuan Cancer Center, School of Medicine, University of Electronic Science and Technology of China, Chengdu, China; 3 Department of Orthodontics, West China Hospital of Stomatology, Sichuan University, Chengdu, China

**Keywords:** Fibroblasts, Periodontal ligament, Hypoxia, Proteome, Pathogenesis of periodontal disease(s)

## Abstract

**Background:**

The periodontal ligament is essential for homeostasis of periodontal tissue. A hypoxic milieu of the periodontal tissue is generated under periodontitis or during orthodontic treatment, which affects the periodontal and bone remodelling process. Here, we provide a comprehensive proteomic characterization of periodontal ligament cells under hypoxic conditions, aiming to reveal previously unappreciated biological changes and to help advance hypoxia-based therapeutic strategies for periodontal diseases.

**Methods:**

Human periodontal ligament cells (hPDLCs) were characterized using immunohistochemistry (IHC) and flow cytometry (FACS). Successful hypoxia treatment of hPDLCs with 1% O_2_ was confirmed by qRT-PCR. Proliferation was evaluated using an MTT assay. The proteomic expression profile under hypoxia was studied with the isobaric tags for relative and absolute quantification (iTRAQ) approach followed by protein identification and bioinformatic analysis, and western blot verification was performed.

**Results:**

The hPDLCs were positive for vimentin, CD73 and CD105 and negative for keratin, CD34 and CD45. After hypoxia treatment, the mRNA expression of hypoxia-inducible factor 1a (*HIF1a)* was upregulated. The proliferation rate was elevated during the first 6 h but decreased from 6 h to 72 h. A total of 220 differentially expressed proteins were quantified in hPDLCs under hypoxia (1% O_2_, 24 h), including 153 upregulated and 67 downregulated proteins, five of which were verified by western blot analysis. The Gene Ontology enriched terms included the energy metabolic process, membrane-bound organelle and vesicle, and protein binding terms. Kyoto Encyclopedia of Genes and Genomes (KEGG) analysis indicated several involved pathways, including glycolysis/gluconeogenesis, biosynthesis of amino acids, the HIF-1 signalling pathway, and focal adhesion. A protein–protein interaction (PPI) network demonstrated the dominant role of autophagy over apoptosis under hypoxia.

**Conclusion:**

The proteomic profile of hPDLCs under hypoxia was mainly related to energy metabolism, autophagy, and responses to stimuli such as adhesion and inflammation. Previously unrecognized proteins including solute carrier family proteins, heat shock proteins, ubiquitination-related enzymes, collagen and S100 family proteins are involved in adaptive response to hypoxia in hPDLCs and are thus of great research interest in future work.

**Electronic supplementary material:**

The online version of this article (10.1186/s12953-019-0151-2) contains supplementary material, which is available to authorized users.

## Background

The periodontal ligament (PDL) is a narrow connective tissue fibre connecting each tooth to the adjacent alveolar bone [1]. It provides anchorage for the tooth and maintains homeostasis of the surrounding tissue [1, 2]. The PDL is composed of cells (e.g., periodontal fibroblasts, periodontal ligament stem cells, and committed osteoblasts) and extracellular components filled with abundant blood vessels, which form an oxygen-enriched periodontal niche crucial for the normal functions of the cells [2, 3].

However, compared with the oxygen tension in in vitro normoxic cultures of 21%, the in vivo physiological oxygen concentration is 4- to 10-fold lower as tested in bone marrow [4]. The oxygen tension in the dental pulp of rats is approximately 3% [5]. An accurate measurement of rodent bone marrow has shown that the average oxygen tension outside the blood vessels is only 1.8% [6]. Under certain conditions, severe hypoxia can develop. For example, PDL tissue is susceptible to hypoxia when chronic inflammation, occlusal trauma or orthodontic force is present [7–9]. Chronic periodontal inflammation in situ severely damages the surrounding vasculature; coupled with massive proliferation of subgingival microorganisms, this inflammation leads to a low-oxygen pathological microenvironment [9]. During orthodontic tooth movement, the applied force is transmitted via the PDL to the surrounding alveolar bone, and the blood flow is reduced at the compressed side, leading to ischaemia that affects PDLC behaviour and the absorption of the compressed alveolar bone [10]. Under circumstances of orthodontic force overload or occlusal trauma, the blood vessels in the PDL have been shown to be temporarily blocked, and thrombi have been shown to form [8, 10, 11], which severely impairs periodontal tissues. Therefore, oxygen tension plays an important role in the homeostasis and pathogenesis of periodontal tissue.

Previous studies have demonstrated the critical role of hypoxia in cell viability, bone remodelling and inflammation of periodontal tissue [12–15]. Nevertheless, different studies have often yielded variable results. For instance, whether hypoxia contributes to bone formation or resorption remains disputed [12–15]. How hypoxia affects the periodontal ligament remains poorly understood, and further clarification is needed regarding the role of hypoxia in periodontal tissue.

High-throughput proteomic screening helps to comprehensively identify proteins involved in certain biological events [16]. In the present study, we used the iTRAQ technique to explore proteomic changes in hPDLCs under hypoxic conditions. The hPDLCs were first obtained and characterized and then subjected to a proliferation assay. Then, comparative proteomic analysis of hPDLCs with and without hypoxia treatment was conducted. Differentially expressed proteins were identified, and subsequent bioinformatic analysis was conducted to elucidate the functions and signalling pathways associated with the altered proteins. Our study provides a proteomic map of PDLC response to hypoxia and identified previously unappreciated biological changes. These results might provide potential molecular targets for further intensive exploration regarding the effect of hypoxia on periodontal health and help advance hypoxia-based therapeutic strategies for periodontal diseases.

## Methods

### Cell culture

The protocols for hPDLC procurement and culture followed the guidelines set by the Institutional Review Board of the West China School of Stomatology, Sichuan University. Informed consent was obtained from patients. hPDLCs were obtained according to previously described methods with minor modifications [17]. Briefly, two premolars extracted for orthodontic purposes from one healthy adolescent at the Department of Oral and Maxillofacial Surgery in our hospital were obtained. The teeth were rinsed with phosphate-buffered saline (PBS, HyClone, Logan, USA) containing 100 U/mL penicillin and 100 mg/mL streptomycin (North China Pharmaceutical Co. Ltd., Shijiazhuang, China). PDL tissues were separated from the middle third of the root surface and then digested with 3 mg/mL collagenase type I (Gibco, Grand Island, NY, USA) and 4 mg/mL dispase (Gibco) for 45 min. The resulting explants were seeded into 35-mm culture dishes containing Minimum Essential Medium α (MEM-α) growth medium supplemented with 10% foetal bovine serum (FBS) (Gibco), 100 U/mL penicillin and 100 mg/mL streptomycin. The dish was incubated at 37 °C in a humidified atmosphere of 5% CO_2_. When they reached 80–90% confluence, the cells were detached using EDTA-trypsin (HyClone) and passaged. All cells were used at passages 3 to 4.

### Characterization of hPDLCs

Flow cytometry was performed for the characterization of hPDLC surface markers. Briefly, hPDLCs were washed with PBS and detached with 0.5% EDTA-trypsin for 2 min, and the reaction was neutralized with serum-containing medium. The suspended cells were centrifuged at 1200 rpm for 5 min, and the supernatant was aspirated. The precipitates were re-suspended in cold PBS, and the suspensions were then centrifuged at 1200 rpm for 5 min. This process was repeated 3 times. The samples were separately labelled with optimal dilutions of fluorescein isothiocyanate-conjugated monoclonal antibodies against CD34, CD45, CD73 and CD105 at 4 °C in the dark. After 20 min of incubation, the cells were washed with cold PBS containing 1% BSA and then analysed with a flow cytometer (Beckman Coulter, US). Nonspecific fluorescence was determined by incubating cells with isotype-matched conjugated mAbs. The data were analysed using CellQuest software.

The expression of keratin and vimentin in hPDLCs was then characterized by immunostaining. Briefly, hPDLCs were rinsed with PBS for 3 times and fixed in 4% paraformaldehyde for 20 min. The fixed cells were treated with 0.5% Triton X-100 and 3% hydrogen peroxide for 15 min. The cells were then incubated for 1 h at 37 °C with a primary mouse anti-pan-cytokeratin antibody (1:500) and a primary rabbit anti-vimentin antibody (1:500) (Abcam, Cambridge, MA, USA). Thereafter, the cells were washed with PBS for 3 times and incubated with corresponding secondary antibodies for 30 min at 37 °C. Finally, the cells were stained with a DAB kit (ZSGB-BIO, Beijing, China) and haematoxylin and viewed under an inverted microscope (Olympus, IX71, Tokyo, Japan).

### Proliferation of hPDLCs

hPDLCs at passage 4 were seeded into 96-well plates at 20% O_2_. After 12 h, the cells were transferred to a tri-gas incubator with 1% O_2_ at 37 °C to continue culturing. At a predetermined time, 10 μL of MTT (Aladdin, Shanghai, China) (5 mg/mL) was added to each well (final concentration 0.5 mg/mL), and the cells were further incubated for 3 h. The formed formazan in each well was dissolved in 150 μL of DMSO, and the absorbance was measured at 570 nm. To validate the MTT results, direct cell counting was performed in 24-well plate with automated cell counter (TC20™, BIORAD, California, United States).

### Quantitative RT-PCR

Total RNA from cultured hPDLCs was extracted using TRIzol reagent (Invitrogen) according to the manufacturer’s instructions. The total RNA was reverse transcribed into cDNA using a PrimeScript RT Reagent Kit with gDNA Eraser (Takara). The RNA purity and concentration were measured using a NanoDrop 2000 spectrophotometer (Thermo Fisher Scientific). Hypoxia-inducible factor 1a (HIF1a) was selected as a marker for hypoxic conditions. qRT-PCR was performed in a 20 μL reaction system with 40 cycles in a CFX96 Real-Time System (Bio-Rad). β-Actin was used as an internal control. All experiments were performed in triplicate, and the results were evaluated using the 2^-ΔΔCt^ method.

### Protein extraction and purification

A total of 6 samples were prepared. Cells were seeded into 6-well plates and pre-cultured for 12 h for adhesion. Then, three cell samples were moved to a tri-gas incubator (Thermo Fisher Scientific, Waltham, MA, USA), and culturing was continued at 37 °C with 1% O_2_ and 5% CO_2_ for another 24 h. The other three cell samples were set as control groups and were cultured at 37 °C with 20% O_2_ and 5% CO_2_ for 24 h. Total protein extraction was then performed using a lysis solution followed by subsequent ultrasonication and centrifugation. The supernatant was collected, and the concentration was determined by the BCA method. The samples were then stored at − 80 °C for isobaric tags for relative and absolute quantitation analysis.

### Isobaric tags for relative and absolute quantification (iTRAQ) analysis and protein identification

SDS-PAGE electrophoresis was first carried out for protein quantification. The protein samples were then reduced, cysteine-blocked and digested, and protein labelling and mass spectrometry (MS) analysis were performed. Two-dimensional liquid chromatography-mass spectrometry (2D-LC-MSMS) analysis including reversed-phase chromatographic separation (Agilent Technologies, Santa Clara, CA, USA) and reversed-phase chromatography on a TripleTOF (AB SCIEX, USA) was conducted.

### Gene ontology and KEGG pathway analyses

Gene Ontology (GO) analyses was employed to illustrate the attributes of the genes and gene products. Generally, GO annotation reveals the cellular components, molecular functions and biological processes associated with gene products. The potential biological pathways involved in hypoxia were also analysed based on the Kyoto Encyclopedia of Genes and Genomes (KEGG) database. The significance of the GO term and KEGG enrichment results was denoted by *P* values. Statistical significance was set at *P* < 0.05.

### Protein–protein interaction (PPI) network

A protein-protein interaction network was constructed based on the STRING database and Cytoscape software. Proteins enriched in autophagy and apoptosis were chosen to draw the network.

### Western blot analysis

The extracted protein samples were electrophoresed via 12% or 15% SDS-polyacrylamide gels according to their molecular weights and then transferred to PVDF membranes. The membranes were blocked with 5% skim milk for 1 h and incubated with rabbit polyclonal anti-MIF (Proteintech 20,415–1-A, 1:1000), rabbit polyclonal anti-S100A10 (Proteintech 11,250–1-AP, 1:1000), rabbit-polyclonal anti-S100A9 (Proteintech, 26,992–1-AP, 1:1000), rabbit polyclonal anti-LDHA (Proteintech 19,987–1-AP, 1:2000) and rabbit polyclonal anti-GAPDH (Proteintech 10,494–1-AP, 1:5000) primary antibodies at 4 °C overnight before being incubated with HRP-conjugated goat anti-rabbit IgG secondary antibodies (Cell Signalling L3012–1, 1:5000). The membranes were finally visualized using Immobilon reagents (Millipore).

### Statistical analysis

The protein data for identification and quantification were processed with Protein Pilot Software v. 5.0 (AB SCIEX, USA) against the *Homo sapiens* database using the Paragon algorithm. Peptides in the 95% confidence interval were selected, and each confident protein was identified and confirmed with at least one unique peptide. Only proteins with fold changes > 1.2 or < 5/6 were considered differentially expressed. Annotations of identified proteins were done according to GO (http://www.geneontology.org/). Pathways enrichment was performed with KEGG database. PPI network was constructed based on the STRING database. The Benjamin-Hochberg FDR correction was used for multiple comparison and only corrected values at *P* < 0.05 were considered significant. Cell proliferation is expressed as the mean ± standard deviation of three independent experiments and was analysed using SPSS 21.0 software. Student’s t-test was used to calculate the significance of differences between groups. *P* < 0.05 was considered to indicate statistical significance.

## Results

### Characterization of hPDLCs

**Fig. 1:**
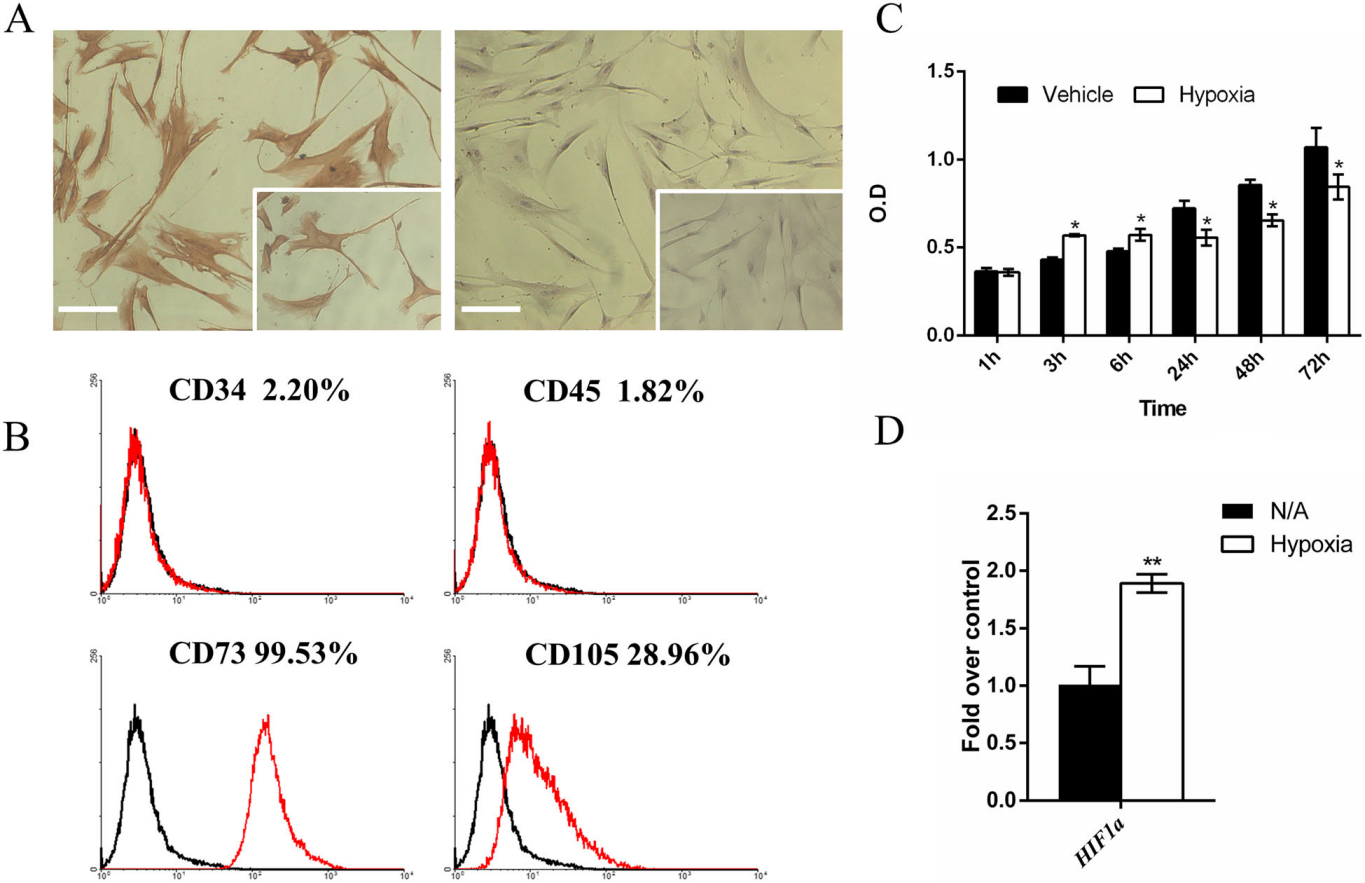
**a** Characterization of hPDLCs. Immunohistochemical staining showed the expression of vimentin in the cytoplasm (A, left), but keratin was not found (A, right). The insert shows a higher magnification of 200X. Scale bar, 100 μm. **b** The flow cytometry results showed that hPDLCs expressed mesenchymal stem cell-associated surface markers (CD73 and CD105) but did not express hematopoietic surface markers (CD34 and CD45). **c** Proliferation rates of hPDLCs. During the first 6 h, the proliferation rates of hPDLCs under hypoxia were higher than those of hPDLCs under normoxia. From 24 h to 72 h, the proliferation rates under hypoxia decreased. The values are presented as the mean ± SD (*Student’s t-test, *P* < 0.05). **d** hPDLCs under hypoxia expressed higher levels of *HIF1a* mRNA than control group hPDLCs

Cells adherent to the flask showed a fibroblast-like spindle shape, and immunohistochemical staining showed positive expression of the fibroblast marker vimentin in the cytoplasm and negative expression of keratin (Fig. 1a). Flow cytometry analysis showed that hPDLCs exhibited mesenchymal-derived stem cell-associated surface markers (CD73 and CD105) and did not express haematopoietic surface markers (CD34 and CD45) (Fig. 1b). Thus, consistent with a previous report, hPDLCs are mesoderm-derived fibroblasts.

### The proliferation of hPDLCs under hypoxia

Hypoxia affected the proliferation rates of hPDLCs. During the first 6 h, the proliferation rates of hPDLCs in 1% O_2_ were higher than those of hPDLCs in normoxia. However, from 24 h to 72 h, the proliferation rates of hPDLCs in 1% O_2_ slowed and became lower than those of cells in the control group (Fig. 1c). The result of direct cell counting is in accordance with the MTT results Additional file 1.

### qRT-PCR verification of hypoxic conditions

qRT-PCR was performed to verify the establishment of hypoxic conditions. After 48 h of hypoxia treatment, hPDLCs showed elevated expression of *HIF1a*, which is a canonical hypoxia marker (Fig. 1d). This result verifies the establishment of hypoxic conditions.

### Proteomic profiles of hPDLCs under hypoxia

**Fig. 2:**
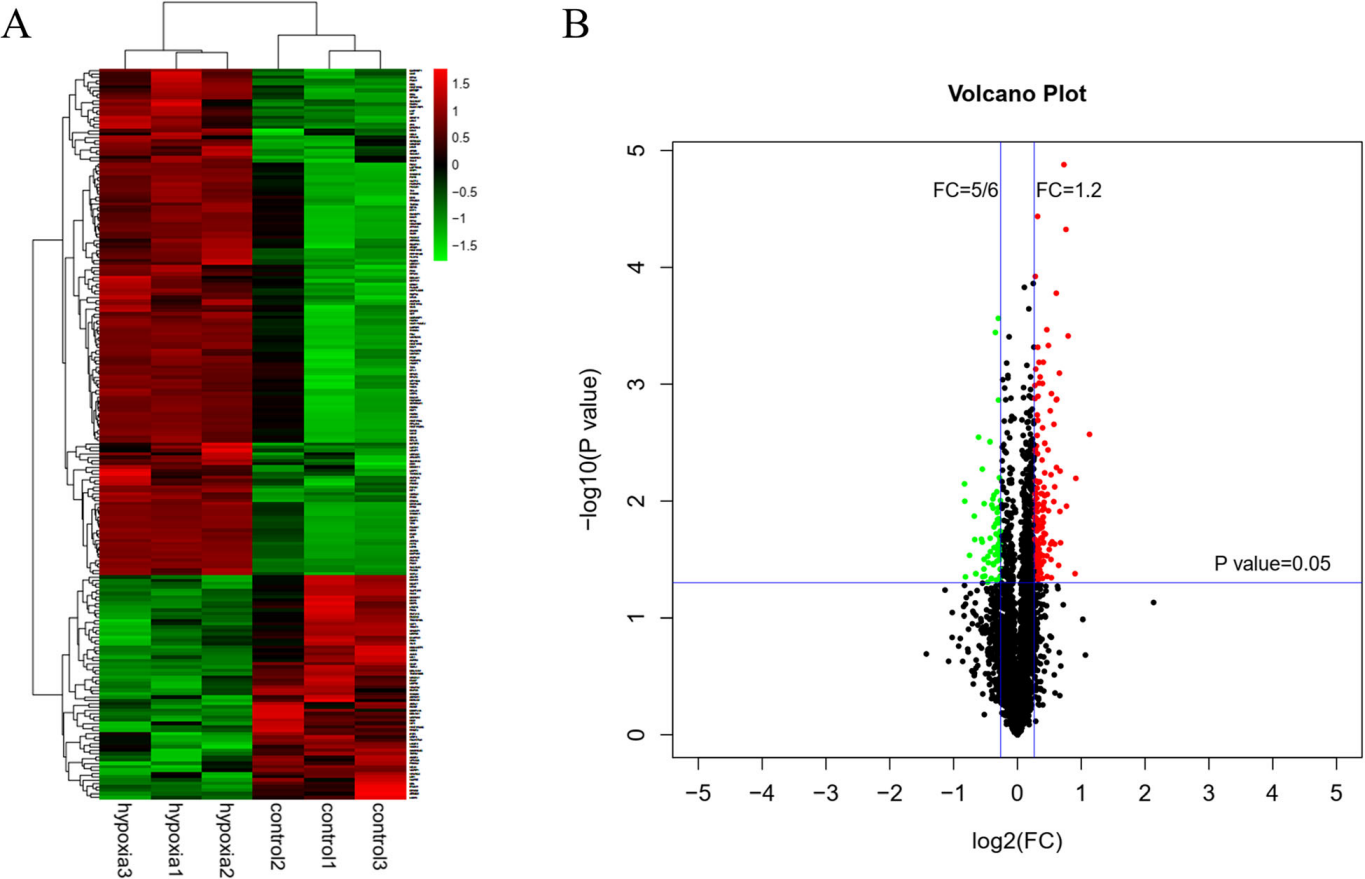
Overview of iTRAQ proteomic analysis. **a** Hierarchical clustering of the differentially expressed proteins. The heat map demonstrates that expression patterns were altered under hypoxia. Red denotes high relative expression, and green denotes low relative expression. **b** The differentially expressed proteins according to fold changes (FCs) and *P* values are depicted with a volcano plot. Proteins with a *P* < 0.05 and a FC < 5/6 or >1.2 were considered to be significantly differentially expressed. Red dots denote upregulated proteins, and green dots denote downregulated proteins

The proteomic profiles of hPDLCs under hypoxia were identified. A total of 3977 proteins were quantified in hPDLCs in all six samples (*n* = 3 for each group). Among these proteins, 220 proteins were differentially expressed under hypoxia with statistical significance, including 153 upregulated proteins and 67 downregulated proteins, as demonstrated by heat map analysis (Fig. 2a). The volcano plot displays these differentially expressed proteins according to their fold changes and *P* values (Fig. 2b). All the altered proteins are accessible in Additional file 1: Table S1.

### Functional categories

**Fig. 3:**
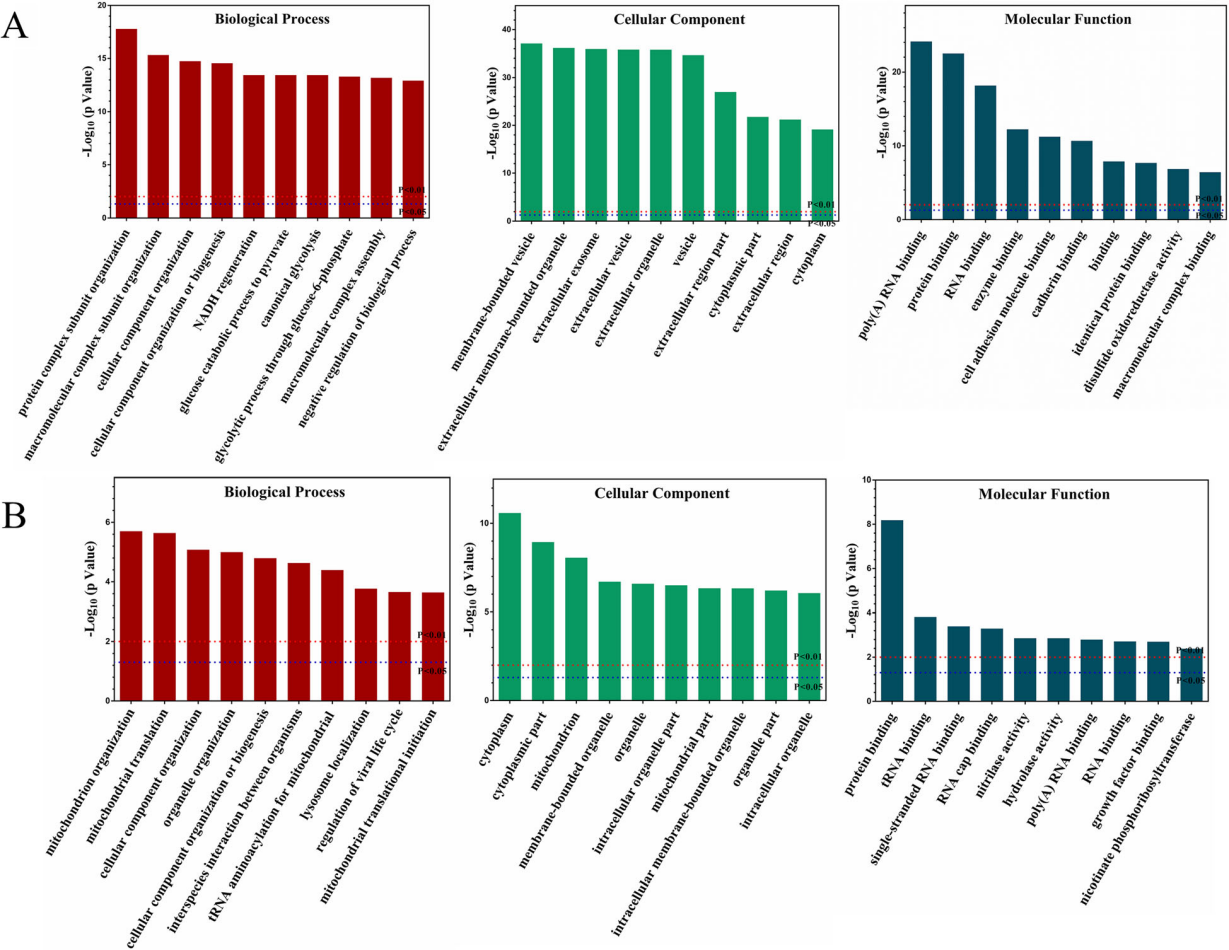
Enrichment analysis of GO terms for the differentially expressed proteins. The upregulated (**a**) and downregulated (**b**) protein terms in the biological process, cellular component and molecular function categories are depicted

Gene Ontology enrichment analysis of the altered proteins revealed that most of the enriched terms were related to energy metabolic processes, membrane-bound organelles and vesicles, protein binding and mitochondrial organization (Fig. 3). In detail, biological processes such as cell component organization, NADH regeneration and mitochondrial functions were altered. Cellular components such as membrane-bound vesicles and extracellular exosomes were changed. Molecular functions such as poly(A) RNA binding, protein binding and cadherin binding were associated with the differentially expressed proteins.

### Relative pathway and protein–protein interaction analysis

**Fig. 4:**
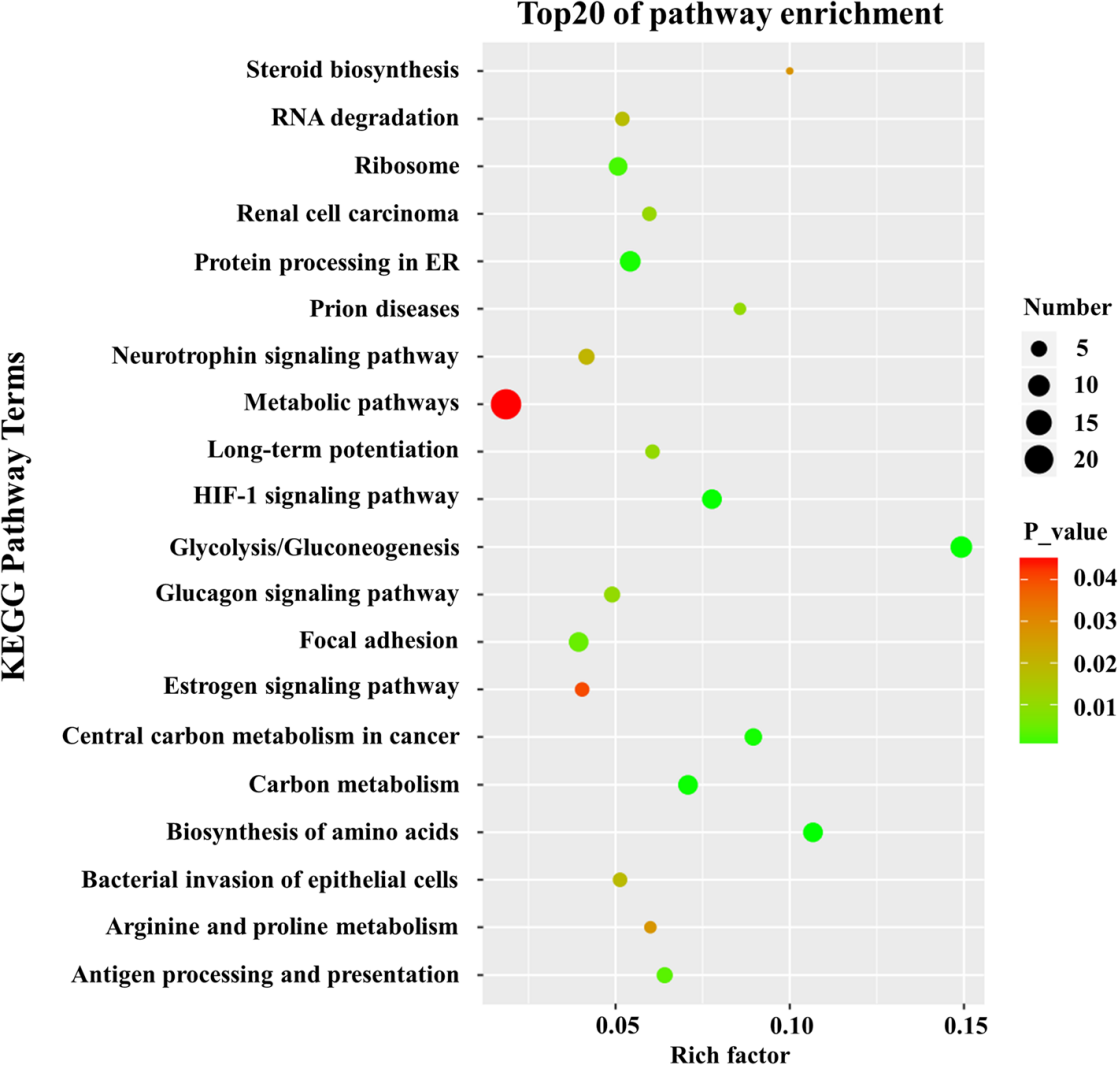
Enrichment analysis of KEGG pathways for the differentially expressed proteins. The bubble chart depicts the top 20 enriched pathways. The colour of each dot denotes the *P* value, and the size of each dot denotes the number of differentially expressed proteins

**Fig. 5:**
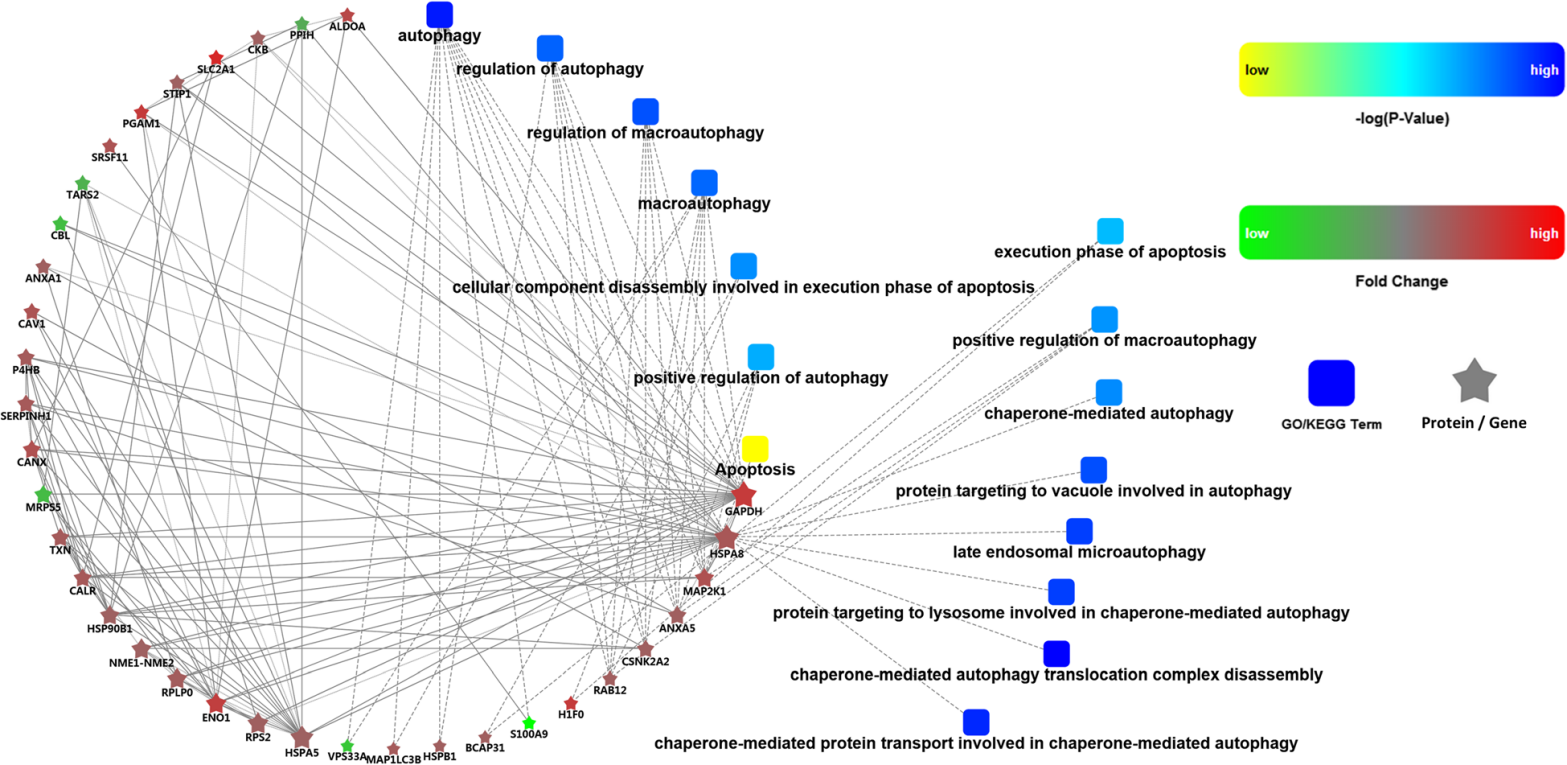
Protein–protein interaction network. Proteins related to apoptosis and autophagy were chosen for analysis

A total of 21 KEGG pathway terms were enriched with statistical significance (Fig. 4). The ten KEGG pathway terms included 1) glycolysis/gluconeogenesis, 2) biosynthesis of amino acids, 3) the HIF-1 signalling pathway, 4) carbon metabolism, 5) central carbon metabolism in cancer, 6) protein processing in the endoplasmic reticulum, 7) ribosomes, 8) antigen processing and presentation, 9) focal adhesion, and 10) prion diseases. Furthermore, proteins related to cell death were chosen, and a protein–protein interaction network was constructed (Fig. 5). The network illustrated the dominant role played by autophagy under hypoxia and identified potential proteins that mediated autophagy and apoptosis, such as heat shock 70 kDa protein 8 (HSPA8), dual specificity mitogen-activated protein kinase kinase 1 (MAP2K1), Annexin A5 (ANXA5), and S100 calcium-binding protein A9 (S100A9).

### Western blot validation of differentially expressed proteins

**Fig. 6:**
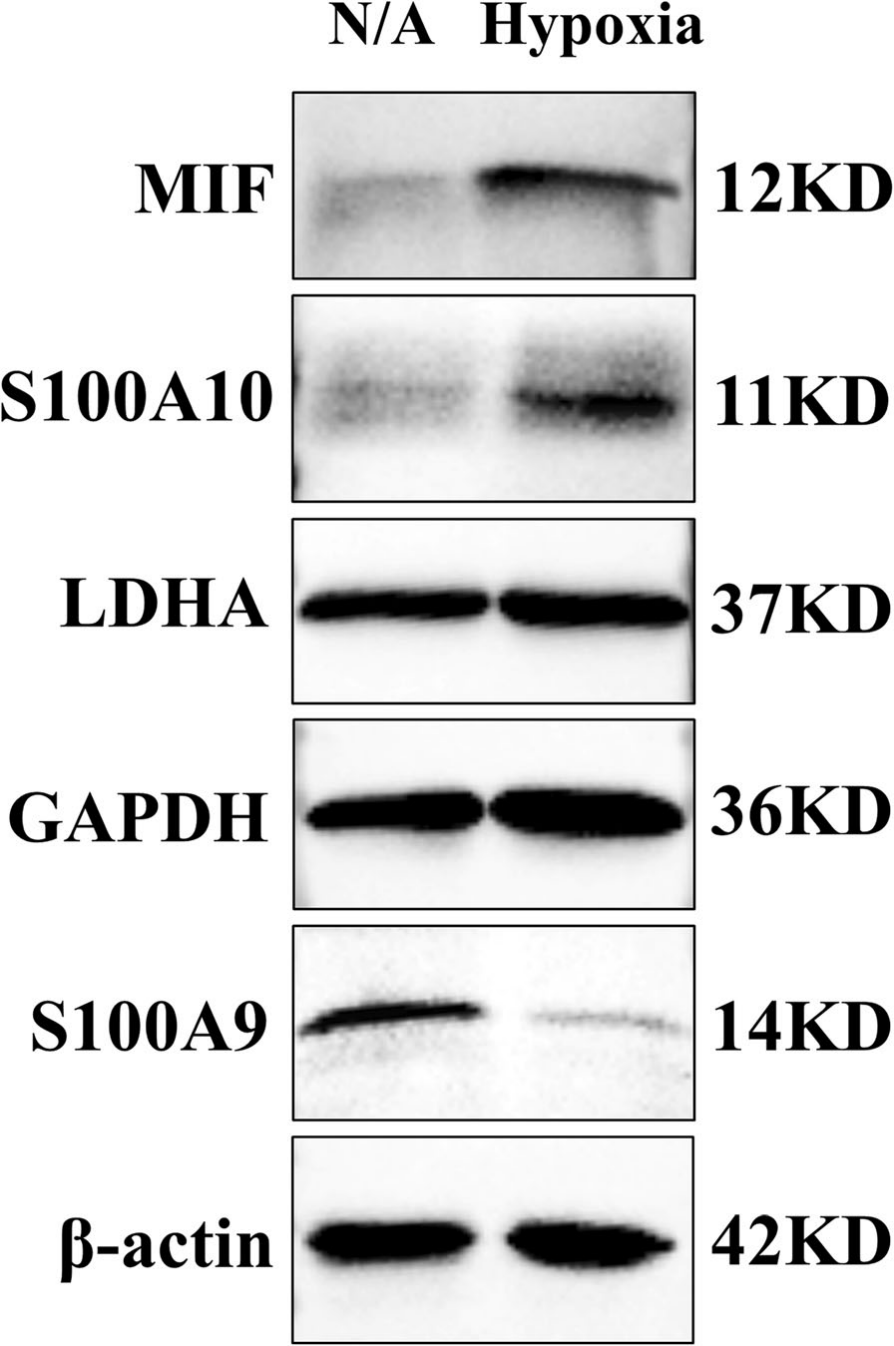
Western blot verification of the iTRAQ analysis results. The protein expression of MIF, S100A10, LDHA, and GAPDH was upregulated, while that of S100A9 was downregulated, consistent with the iTRAQ results

A total of 220 proteins were identified as differentially expressed by iTRAQ-based analysis. Five proteins, including macrophage migration inhibitory factor (MIF), lactate dehydrogenase A (LDHA), S100A10, glyceraldehyde-3-phosphate dehydrogenase (GAPDH) and S100A9, were chosen to be verified by western blot analysis (Fig. 6). MIF, LDHA, S100A10 and GAPDH were upregulated and S100A9 was downregulated in the hypoxia group compared with the control groups, which is consistent with the results of iTRAQ analysis.

## Discussion

Oxygen concentration is a crucial regulating factor affecting physiological processes and pathogeneses throughout the human body during the entire life span [18–20], including in periodontal tissue [7–9]. However, there is a lack of consensus on the exact effects of hypoxia on periodontal homeostasis and pathogenesis due to a lack of in-depth mechanistic studies analysing the effects of distinct experimental variables, including oxygen tension, treatment time, and the presence or absence of differentiation induction. Here, in our work, we aimed to identify potential molecular targets for further intensive exploration regarding the effect of hypoxia on periodontal health. We chose a generally recognized condition, 1% O_2_ for 24 h without additional induction, to perform the experiment [12, 13]. Specifically, 1% O_2_ was chosen rather than 2% or 5% O_2_ because we sought to replicate pathogenic and pathological periodontal conditions contributing to hypoxia, which reciprocally enhances the disease process through a series of biological responses.

Cell proliferation status under 1% O_2_ was studied first, and the results were partially in accordance with the findings of previous studies [12–15]. Wu et al. studied the viability of periodontal ligament stem cells (PDLSCs) under 2% O_2_ and showed that hypoxia promoted cell proliferation in the first 24 h [15]. Zhang et al. explored the effects of different concentrations (21, 5, and 1% O_2_) on PDLC proliferation and showed that higher levels of hypoxia increased growth during the first 24 h but markedly restrained it at 48 h and 72 h [13]. It seems that exposing cells to severe or prolonged hypoxia can induce cell death, while exposure to mild or temporally restrained hypoxia can stimulate a cell adaptive response.

A previous study revealed that protein expression and cell function were greatly modulated in response to hypoxia [4, 18]. Analysis of the proteomic profile demonstrated changes in three main processes in hypoxia-treated hPDLCs: energy metabolism, autophagy, and adaptive responses to stimuli such as adhesion and inflammation.

As expected, energy metabolism changed under hypoxia. Both the GO and KEGG pathway analyses revealed abundant enrichment of terms for energy metabolism. A large number of differentially expressed proteins related to cell metabolism were identified, including glyceraldehyde-3-phosphate dehydrogenase (GAPDH), lactate dehydrogenase A (LDHA), phosphoglycerate kinase (PGK1), and phosphoglycerate mutase 1 (PGAM1) [21–23]. Members of the solute carrier (SLC) family, which facilitates zinc and glucose transport, were also upregulated, including SLC39A7, SLC38A2, SLC16A3 and SLC2A1 [24]. These metabolic alterations provide energy essential for cell adaptive responses and partially explain why cell proliferation changed. Importantly, a close relationship between glucose and periodontitis has been widely recognized, but whether and how hypoxia contributes to periodontal lesion initiation and progression by altering intracellular glucose metabolism is unclear. Therefore, the interplay among energy metabolism-associated proteins and their association with inflammation requires further elucidation, especially regarding the relatively understudied SLC family [24].

Apart from energy metabolism, GO analysis revealed abundant enrichment of terms for intracellular and extracellular membrane-bound vesicles and organelles. Intracellularly, enhanced modification of cytoplasmic components under hypoxia is indicative of active apoptosis or autophagy [25, 26]. To illustrate protein associations, a PPI network between apoptosis and autophagy was constructed. Interestingly, under severe hypoxia, autophagy plays a dominant role over apoptosis. The network revealed elevated expression of the heat shock protein (HSP) family, which exerts a cytoprotective function against stress [27]. Heat shock 70 kDa protein 8 (HSPA8), which was at the centre of the network, acts as a crucial component of chaperone-mediated autophagy (CMA), by which proteins are imported into lysosomes for degradation [27, 28]. HSPA8 extensively interacted with proteins including HSP beta 1 (HSPB1) and heat shock 70 kDa protein 5 (HSPA5), synergistically exerting anti-apoptotic effects on hPDLCs. Additionally, HSPs modulate cell development, differentiation and inflammatory processes [29]. Several studies have indicated the involvement of HSP 60 in tooth development and periodontitis [30–32]. However, whether HSP 70 contributes to periodontal inflammation or remodelling remains poorly explored [33]. Thus, it is of great research interest to study the role of HSP 70 in PDLC adaptive responses to external stress, such as hypoxia and pathogen invasion. In addition, the expression of ubiquitin-related enzymes was altered under hypoxia, including ubiquitin-conjugating enzyme E2 (UB2G1), ubiquitin-associated protein 1 (UBAP1), and ubiquitin-like protein 5 (UBL5). These proteins target substrates for proteasome degradation and might contribute to autophagy [34]. Furthermore, the expression of apoptosis repressor nucleolar protein 3 (NOL3) was increased under hypoxia [35]. The hypoxia inducible factor-1 (HIF-1) signalling pathway was also activated, which is considered a protective pathway against apoptosis in mesenchymal stem cells [36, 37]. These data demonstrate that instead of apoptosis, autophagy might play a decisive role in the hPDLC response to hypoxia.

The cellular response to hypoxia involved more than energy metabolism and autophagy. Cell components related to adhesion were also changed. Focal adhesion was enriched in KEGG pathway analysis; among the associated proteins, collagen alpha-1(II) chain (COL2A1), caveolin 1 (CAV1), caveolin 2 (CAV2), Ras-related protein Rap-1a (RAP1A), Ras-related protein Rap-1b (RAP1B) and MAP 2 K were upregulated, while (COL1A1) and Integrin alpha-11 (ITGA11) were downregulated. One of the unique features of PDL is that this unmineralized soft tissue connects mineralized cementum and alveolar bone; to fulfil this function, abundant cell–cell and cell–matrix interactions are indispensable. Therefore, we inferred that enhanced focal adhesion under hypoxia might be a result of an adaptive response to maintain the periodontium structure. Intriguingly, COL1A1 and COL12A1, the main collagen fibres composing the periodontal ligament, were downregulated under hypoxia [38]. However, COL2A1, the major cartilaginous extracellular matrix (ECM) molecule, was upregulated under hypoxia [39]. Type II collagen is known to regulate chondrogenic differentiation of mesenchymal stem cells (MSCs), but no evidence has ever linked it to the periodontal ligament. Accordingly, further study might focus on the effect of COL2A1 on the PDL. At the same time, alleviation of the hypoxia in the context of periodontitis to promote collagen biosynthesis and alignment might be exploited for PDL regeneration and damage repair in future work.

Development of hypoxia-based treatment strategies might contribute to periodontal health. For example, prolyl hydroxylase (PHD) inhibitors can stabilize HIF1a and mimic the hypoxic condition. One study showed that PHD inhibitors dimethyloxallyl glycine, desferrioxamine, l-mimosine and CoCl_2_ can induce the production of VEGF in PDLCs, which promotes bone regeneration [40]. Hypoxia has been shown to correlate closely with inflammation, angiogenesis and osteogenesis [15, 20, 41]. Therefore, identifying and confirming whether these effects also applied to hPDLCs under severe hypoxia has clinical significance. A previous study demonstrated that hypoxia induced the mRNA expression of VEGF and IL-6 and the paracrine secretion of prostaglandin E2 (PGE_2_) in PDLCs [12, 15]. In this work, we identified that inflammation-related proteins such as CAV2 and macrophage migration inhibitory factor (MIF) were increased [42, 43]. S100A4 was also upregulated and enriched for the receptor for advanced glycation end products (RAGE) pathway, which plays a significant role in inflammatory processes and chronic diseases [44]. However, there is insufficient evidence to conclude that the inflammatory response was activated in hPDLCs in response to hypoxia. We found that the protein S100A9, which is actively involved in the NF-κB signalling pathway, was the most downregulated among the 67 decreased proteins [45]. Neither GO nor KEGG pathway analysis revealed enriched terms related to inflammation. Thus, whether the inflammatory response was triggered in hPDLCs exposed to hypoxia remains to be elucidated. Regarding angiogenesis and osteogenesis, few terms were enriched in the GO and KEGG pathways. We suppose this result is reasonable since the cell culture medium for the hPDLCs in the present work only consisted of essential medium without any inducing factors. The absence of expression changes in proteins related to angiogenesis and osteogenesis under these culture conditions is not sufficient to refute a potential effect of hypoxia on these proteins, considering that these effects might take place in the context of certain environmental cues.

Finally, we noticed great alteration of the S100 protein family under hypoxia. Among 21 types of S100 proteins, 5 were differentially expressed under hypoxia; S100A4, S100A6, S100A10, and S100A11 were elevated, and S100A9 was reduced. Surprisingly, a recent study analysing the proteomic expression profiles of PDLSCs and dental pulp stem cells (DPSCs) demonstrated a similar result in which higher expression of S100A4, S100A10 and S100A11 but lower expression of S100A9 occurred in PDLSCs upon hypoxia exposure [46]. As S100 family proteins are most known as calcium-binding proteins with functions in cell survival, cytoskeletal dynamics and differentiation, further study regarding their roles in periodontal remodelling is needed, especially regarding the interplay among the S100A4, S100A9, S100A10 and S100A11 subfamily proteins in both dental pulp and PDL tissue [47].

As our proteomic analysis was based on PDLCs from one single donor, the impact of biological variation among different donors is greatly reduced. However, it increases the sample selection bias and might cause false results, though the identified proteins were partially validated by western blot. Further studies based on different individuals are expected. Besides, PDLCs is cultured with essential medium without inducing factors in the present study. Combining factors such as osteogenic cues and microorganism with hypoxia into analyses is of great clinical significance considering their critical role in periodontal health.

## Conclusion

In conclusion, we have illustrated, for the first time, the proteomic changes in hPDLCs under hypoxic conditions. We identified 220 differentially expressed proteins under hypoxia, and further bioinformatic analysis indicated that these proteins were closely correlated with energy metabolism, autophagy and adaptive responses to stimuli such as adhesion and inflammation. Our results provide useful resources for further study on the underlying mechanisms of the effects of hypoxia on hPDLC behaviour and periodontal tissue remodelling.

## Additional file

Additional file 1: **Table S1.** List of 220 differentially expressed proteins in hypoxia-treated hPDLCs with Uniprot accession, gene symbol, protein description, expression fold change and *P* value. **Figure S1.** Direct cell counting of hPDLCs number after hypoxic treatment (*n* = 3). Data are represented as mean ± SEM,; ^*^P < 0.05, ^**^P < 0.01 by two-tailed Student’s t test. (DOCX 110 kb)

## Data Availability

Differentially expressed proteins list is available in Additional file 1: Table S1.

## Abbreviations

2D-LC-MSMS: Two dimensional liquid chromatography-mass spectrometry
ANXA5: Annexin A5
CAV1: Caveolin 1
CAV2: Caveolin 2
CMA: Chaperone-mediated autophagy
COL2A1: Collagen alpha-1(II) chain
DPSCs: Dental pulp stem cells
FACS: Flow cytometry
FBS: Fetal bovine serum
GAPDH: Glyceraldehyde-3-phosphate dehydrogenase
GO: Gene ontology
HIF-1: Hypoxia inducible factor-1
hPDLCs: Human periodontal ligament cells
HSP: Heat shock protein
HSPA8: Heat shock 70 kDa protein 8
HSPB1: HSP beta 1
IHC: Immunohistochemistry staining
ITGA11: Integrin alpha-11
iTRAQ: Isobaric tags for relative and absolute quantification
KEGG: Kyoto Encyclopedia of Genes and Genomes
LDHA: Lactate dehydrogenase A
MAP 2 K1: Mitogen-activated protein kinase kinase 1
MEM-α: Minimum Essential Medium α
MIF: Macrophage migration inhibitory factor
MS: Mass spectrometry
MSCs: Mesenchymal stem cells
NOL3: Nucleolar protein 3
PDL: Periodontal ligament
PDLSCs: Periodontal ligament stem cells
PGAM1: Phosphoglycerate mutase 1
PGE2: Prostaglandin E2
PGK1: Phosphoglycerate kinase
PPI: Protein-protein interaction
RAGE: Advanced glycation end products
RAP1A: Ras-related protein Rap-1a
RAP1B: Ras-related protein Rap-1b
S100A9: S100 calcium-binding protein A9
SLC: Solute carrier
UB2G1: Ubiquitin-conjugating enzyme E2
UBAP1: Ubiquitin-associated protein 1
UBL5: Ubiquitin-like protein 5
