# Supplementary material for: Proteomic analysis of human periodontal ligament cells under hypoxia
Source: Proteome Sci. 2019 Aug 31;17:3. doi: 10.1186/s12953-019-0151-2 (PMC6717648; doi:10.1186/s12953-019-0151-2)
Supplement: Supplementary file 1 — Table S1. List of 220 differentially expressed proteins in hypoxia-treated hPDLCs with Uniprot accession, gene symbol, protein description, expression fold change and P value. Figure S1. Direct cell counting of hPDLCs number after hypoxic treatment (n = 3). Data are represented as mean ± SEM,; *P < 0.05, **P < 0.01 by two-tailed Student’s t test. (DOCX 110 kb) [file 12953_2019_151_MOESM1_ESM.docx]

Additional file 1

**Table S1** List of 220 differentially expressed proteins in hypoxia-treated hPDLCs with Uniprot accession, gene symbol, protein description, expression fold change and P value.

| **Accession^#^** | **Gene** | **Description** | **FC** | **P value** |
| --- | --- | --- | --- | --- |
| P10412 | HIST1H1E | Histone H1.4 | 2.19 | 0.003 |
| Q6WKZ4 | RAB11FIP1 | Rab11 family-interacting protein 1 | 1.89 | 0.006 |
| H3BUP2 | NOL3 | Nucleolar protein 3 (Fragment) | 1.87 | 0.042 |
| P00338 | LDHA | L-lactate dehydrogenase A chain | 1.73 | 0.000 |
| P11166 | SLC2A1 | Solute carrier family 2, facilitated glucose transporter member 1 | 1.70 | 0.011 |
| O15427 | SLC16A3 | Monocarboxylate transporter 4 | 1.69 | 0.000 |
| P00558 | PGK1 | Phosphoglycerate kinase 1 | 1.66 | 0.000 |
| P16403 | HIST1H1C | Histone H1.2 | 1.60 | 0.023 |
| P60903 | S100A10 | Protein S100-A10 | 1.59 | 0.006 |
| Q5T6W2 | HNRNPK | Heterogeneous nuclear ribonucleoprotein K (Fragment) | 1.59 | 0.012 |
| P14174 | MIF | Macrophage migration inhibitory factor | 1.58 | 0.001 |
| P62253 | UBE2G1 | Ubiquitin-conjugating enzyme E2 G1 | 1.56 | 0.036 |
| P60174 | TPI1 | Triosephosphate isomerase | 1.53 | 0.001 |
| P04406 | GAPDH | Glyceraldehyde-3-phosphate dehydrogenase | 1.53 | 0.005 |
| P18669 | PGAM1 | Phosphoglycerate mutase 1 | 1.52 | 0.001 |
| P07305 | H1F0 | Histone H1.0 | 1.52 | 0.000 |
| P51784 | USP11 | Ubiquitin carboxyl-terminal hydrolase 11 | 1.50 | 0.023 |
| P26447 | S100A4 | Protein S100-A4 | 1.50 | 0.008 |
| P06733 | ENO1 | Alpha-enolase | 1.49 | 0.002 |
| P17096 | HMGA1 | High mobility group protein HMG-I/HMG-Y | 1.48 | 0.010 |
| P51636 | CAV2 | Caveolin-2 | 1.46 | 0.022 |
| G8JLH6 | CD9 | Tetraspanin (Fragment) | 1.45 | 0.001 |
| Q9NZ09 | UBAP1 | Ubiquitin-associated protein 1 | 1.44 | 0.045 |
| O14561 | NDUFAB1 | Acyl carrier protein, mitochondrial | 1.44 | 0.032 |
| P62834 | RAP1A | Ras-related protein Rap-1A | 1.44 | 0.006 |
| U3KQK0 | HIST1H2BN | Histone H2B | 1.43 | 0.023 |
| A0A0A0MTS2 | GPI | Glucose-6-phosphate isomerase (Fragment) | 1.43 | 0.002 |
| Q9P1F3 | ABRACL | Costars family protein ABRACL | 1.41 | 0.026 |
| P29966 | MARCKS | Myristoylated alanine-rich C-kinase substrate | 1.40 | 0.012 |
| P04075 | ALDOA | Fructose-bisphosphate aldolase A | 1.40 | 0.000 |
| P31949 | S100A11 | Protein S100-A11 | 1.39 | 0.004 |
| P27144 | AK4 | Adenylate kinase 4, mitochondrial | 1.38 | 0.009 |
| S4R417 | RPS15 | 40S ribosomal protein S15 | 1.38 | 0.044 |
| E7ETZ0 | CALM1 | Calmodulin | 1.38 | 0.029 |
| Q9BTT0 | ANP32E | Acidic leucine-rich nuclear phosphoprotein 32 family member E | 1.38 | 0.000 |
| P03905 | MT-ND4 | NADH-ubiquinone oxidoreductase chain 4 | 1.35 | 0.019 |
| Q9Y2V2 | CARHSP1 | Calcium-regulated heat-stable protein 1 | 1.35 | 0.003 |
| A0A087X2B5 | BSG | Basigin (Fragment) | 1.34 | 0.006 |
| P61970 | NUTF2 | Nuclear transport factor 2 | 1.34 | 0.009 |
| P16401 | HIST1H1B | Histone H1.5 | 1.34 | 0.012 |
| P27824 | CANX | Calnexin | 1.34 | 0.010 |
| M0R1I2 | PLAUR | Urokinase plasminogen activator surface receptor | 1.33 | 0.019 |
| E9PR30 | FAU | 40S ribosomal protein S30 | 1.33 | 0.008 |
| Q02750 | MAP2K1 | Dual specificity mitogen-activated protein kinase kinase 1 | 1.33 | 0.033 |
| P17936 | IGFBP3 | Insulin-like growth factor-binding protein 3 | 1.33 | 0.023 |
| P62750 | RPL23A | 60S ribosomal protein L23a | 1.33 | 0.023 |
| P09104 | ENO2 | Gamma-enolase | 1.32 | 0.001 |
| A0A087WTX2 | SLC39A7 | Zinc transporter SLC39A7 | 1.32 | 0.015 |
| Q9Y223 | GNE | Bifunctional UDP-N-acetylglucosamine 2-epimerase/N-acetylmannosamine kinase | 1.32 | 0.014 |
| P39687 | ANP32A | Acidic leucine-rich nuclear phosphoprotein 32 family member A | 1.32 | 0.011 |
| Q9Y282 | ERGIC3 | Endoplasmic reticulum-Golgi intermediate compartment protein 3 | 1.32 | 0.029 |
| P04183 | TK1 | Thymidine kinase, cytosolic | 1.32 | 0.014 |
| A0A087WUS0 | RPS24 | 40S ribosomal protein S24 | 1.31 | 0.002 |
| O75909 | CCNK | Cyclin-K | 1.31 | 0.046 |
| Q92597 | NDRG1 | Protein NDRG1 | 1.31 | 0.001 |
| Q5T760 | SRSF11 | Serine/arginine-rich-splicing factor 11 (Fragment) | 1.31 | 0.012 |
| A6NIW2 | DOCK11 | Dedicator of cytokinesis protein 11 | 1.31 | 0.021 |
| Q03135 | CAV1 | Caveolin-1 | 1.31 | 0.009 |
| E7EQV9 | RPL15 | Ribosomal protein L15 (Fragment) | 1.31 | 0.017 |
| P09382 | LGALS1 | Galectin-1 | 1.31 | 0.004 |
| P02795 | MT2A | Metallothionein-2 | 1.30 | 0.035 |
| Q15392 | DHCR24 | Delta(24)-sterol reductase | 1.29 | 0.009 |
| P52788 | SMS | Spermine synthase | 1.29 | 0.041 |
| O60869 | EDF1 | Endothelial differentiation-related factor 1 | 1.29 | 0.021 |
| P11142 | HSPA8 | Heat shock cognate 71 kDa protein | 1.28 | 0.028 |
| S4R371 | FABP3 | Fatty acid-binding protein, heart (Fragment) | 1.28 | 0.001 |
| P62805 | HIST1H4A | Histone H4 | 1.28 | 0.017 |
| Q9BZL1 | UBL5 | Ubiquitin-like protein 5 | 1.27 | 0.007 |
| E9PK25 | CFL1 | Cofilin-1 | 1.27 | 0.039 |
| O75688 | PPM1B | Protein phosphatase 1B | 1.27 | 0.031 |
| I3L2J8 | CEP131 | Centrosomal protein of 131 kDa | 1.27 | 0.040 |
| P27797 | CALR | Calreticulin | 1.27 | 0.026 |
| I3L2B2 | EPN2 | Epsin-2 | 1.27 | 0.016 |
| E7ETU9 | PLOD2 | Procollagen-lysine,2-oxoglutarate 5-dioxygenase 2 | 1.27 | 0.001 |
| P50454 | SERPINH1 | Serpin H1 | 1.27 | 0.023 |
| K7EM18 | EIF1 | Eukaryotic translation initiation factor 1 | 1.26 | 0.001 |
| Q14534 | SQLE | Squalene monooxygenase | 1.26 | 0.023 |
| P84090 | ERH | Enhancer of rudimentary homolog | 1.26 | 0.028 |
| P58546 | MTPN | Myotrophin | 1.26 | 0.011 |
| F5GWI9 | CCDC53 | WASH complex subunit CCDC53 | 1.26 | 0.033 |
| P07237 | P4HB | Protein disulfide-isomerase | 1.26 | 0.015 |
| P10599 | TXN | Thioredoxin | 1.25 | 0.047 |
| P61224 | RAP1B | Ras-related protein Rap-1b | 1.25 | 0.036 |
| H3BTL1 | MAP1LC3B | Microtubule-associated protein 1 light chain 3 beta, isoform CRA_f | 1.25 | 0.007 |
| Q02539 | HIST1H1A | Histone H1.1 | 1.25 | 0.016 |
| O15145 | ARPC3 | Actin-related protein 2/3 complex subunit 3 | 1.25 | 0.001 |
| Q15363 | TMED2 | Transmembrane emp24 domain-containing protein 2 | 1.25 | 0.029 |
| Q96C90 | PPP1R14B | Protein phosphatase 1 regulatory subunit 14B | 1.25 | 0.002 |
| A0A0C4DGQ5 | CAPNS1 | Calpain small subunit 1 | 1.25 | 0.000 |
| B2R5W2 | HNRNPC | Heterogeneous nuclear ribonucleoproteins C1/C2 | 1.25 | 0.024 |
| O75506 | HSBP1 | Heat shock factor-binding protein 1 | 1.25 | 0.045 |
| Q13185 | CBX3 | Chromobox protein homolog 3 | 1.25 | 0.025 |
| Q15121 | PEA15 | Astrocytic phosphoprotein PEA-15 | 1.24 | 0.000 |
| P53999 | SUB1 | Activated RNA polymerase II transcriptional coactivator p15 | 1.24 | 0.022 |
| Q96QD8 | SLC38A2 | Sodium-coupled neutral amino acid transporter 2 | 1.24 | 0.004 |
| A8K878 | MANF | Mesencephalic astrocyte-derived neurotrophic factor | 1.24 | 0.012 |
| I3L504 | EIF5A | Eukaryotic translation initiation factor 5A-1 | 1.24 | 0.026 |
| Q9C0E8 | LNP | Protein lunapark | 1.24 | 0.002 |
| Q9H2U1 | DHX36 | ATP-dependent RNA helicase DHX36 | 1.24 | 0.033 |
| Q9NX58 | LYAR | Cell growth-regulating nucleolar protein | 1.24 | 0.008 |
| C9JYN0 | SYPL1 | Synaptophysin-like protein 1 | 1.24 | 0.003 |
| P05388 | RPLP0 | 60S acidic ribosomal protein P0 | 1.24 | 0.015 |
| Q9Y3Y2 | CHTOP | Chromatin target of PRMT1 protein | 1.24 | 0.007 |
| G3V0G1 | GORASP1 | Golgi reassembly stacking protein 1, 65kDa, isoform CRA_d | 1.24 | 0.018 |
| Q13404 | UBE2V1 | Ubiquitin-conjugating enzyme E2 variant 1 | 1.23 | 0.011 |
| P05114 | HMGN1 | Non-histone chromosomal protein HMG-14 | 1.23 | 0.038 |
| P05387 | RPLP2 | 60S acidic ribosomal protein P2 | 1.23 | 0.045 |
| A0A0C4DFV9 | SET | Protein SET | 1.23 | 0.009 |
| P08962 | CD63 | CD63 antigen | 1.23 | 0.003 |
| P62495 | ETF1 | Eukaryotic peptide chain release factor subunit 1 | 1.22 | 0.029 |
| E9PFN5 | GSTK1 | Glutathione S-transferase kappa 1 | 1.22 | 0.014 |
| Q6IQ22 | RAB12 | Ras-related protein Rab-12 | 1.22 | 0.030 |
| A0A087X1Z3 | PSME2 | Proteasome activator complex subunit 2 | 1.22 | 0.016 |
| P14618 | PKM | Pyruvate kinase PKM | 1.22 | 0.018 |
| P04792 | HSPB1 | Heat shock protein beta-1 | 1.22 | 0.009 |
| Q9Y5V3 | MAGED1 | Melanoma-associated antigen D1 | 1.22 | 0.012 |
| Q9H098 | FAM107B | Protein FAM107B | 1.22 | 0.014 |
| Q96HE7 | ERO1A | ERO1-like protein alpha | 1.22 | 0.001 |
| E5RIM7 | ATOX1 | Copper transport protein AT | 1.22 | 0.043 |
| P06703 | S100A6 | Protein S100-A6 | 1.22 | 0.017 |
| P04083 | ANXA1 | Annexin A1 | 1.22 | 0.022 |
| A0A087WTM7 | APOB | Apolipoprotein B-100 | 1.22 | 0.007 |
| P12277 | CKB | Creatine kinase B-type | 1.22 | 0.047 |
| P51572 | BCAP31 | B-cell receptor-associated protein 31 | 1.22 | 0.023 |
| P08758 | ANXA5 | Annexin A5 | 1.22 | 0.024 |
| P19784 | CSNK2A2 | Casein kinase II subunit alpha' | 1.22 | 0.004 |
| Q9NZN4 | EHD2 | EH domain-containing protein 2 | 1.21 | 0.004 |
| P15880 | RPS2 | 40S ribosomal protein S2 | 1.21 | 0.025 |
| F6WQW2 | RANBP1 | Ran-specific GTPase-activating protein | 1.21 | 0.031 |
| P13674 | P4HA1 | Prolyl 4-hydroxylase subunit alpha-1 | 1.21 | 0.000 |
| Q92688 | ANP32B | Acidic leucine-rich nuclear phosphoprotein 32 family member B | 1.21 | 0.022 |
| Q15293 | RCN1 | Reticulocalbin-1 | 1.21 | 0.017 |
| Q09666 | AHNAK | Neuroblast differentiation-associated protein AHNAK | 1.21 | 0.024 |
| P36871 | PGM1 | Phosphoglucomutase-1 | 1.21 | 0.004 |
| Q8TDX7 | NEK7 | Serine/threonine-protein kinase Nek7 | 1.21 | 0.015 |
| Q9NZV1 | CRIM1 | Cysteine-rich motor neuron 1 protein | 1.21 | 0.033 |
| Q32Q12 | NME1-NME2 | Nucleoside diphosphate kinase | 1.21 | 0.009 |
| P31948 | STIP1 | Stress-induced-phosphoprotein 1 | 1.21 | 0.011 |
| P02458 | COL2A1 | Collagen alpha-1(II) chain | 1.21 | 0.040 |
| P48509 | CD151 | CD151 antigen | 1.21 | 0.001 |
| O95881 | TXNDC12 | Thioredoxin domain-containing protein 12 | 1.21 | 0.036 |
| Q13530 | SERINC3 | Serine incorporator 3 | 1.21 | 0.021 |
| P13746 | HLA-A | HLA class I histocompatibility antigen, A-11 alpha chain | 1.21 | 0.001 |
| Q15012 | LAPTM4A | Lysosomal-associated transmembrane protein 4A | 1.21 | 0.014 |
| P61978 | HNRNPK | Heterogeneous nuclear ribonucleoprotein K | 1.21 | 0.010 |
| P39019 | RPS19 | 40S ribosomal protein S19 | 1.21 | 0.033 |
| P14625 | HSP90B1 | Endoplasmin | 1.20 | 0.024 |
| P83731 | RPL24 | 60S ribosomal protein L24 | 1.20 | 0.024 |
| P11021 | HSPA5 | 78 kDa glucose-regulated protein | 1.20 | 0.026 |
| Q6NZI2 | PTRF | Polymerase I and transcript release factor | 1.20 | 0.022 |
| E9PAV3 | NACA | Nascent polypeptide-associated complex subunit alpha, muscle-specific form | 1.20 | 0.032 |
| O75915 | ARL6IP5 | PRA1 family protein 3 | 1.20 | 0.017 |
| Q15366 | PCBP2 | Poly(rC)-binding protein 2 | 1.20 | 0.010 |
| Q96C86 | DCPS | m7GpppX diphosphatase | 0.83 | 0.021 |
| Q969U7 | PSMG2 | Proteasome assembly chaperone 2 | 0.83 | 0.023 |
| Q16626 | MEA1 | Male-enhanced antigen 1 | 0.83 | 0.010 |
| A0A087X0W7 | ACOT2 | Acyl-coenzyme A thioesterase 2, mitochondrial | 0.83 | 0.018 |
| Q8NFV4 | ABHD11 | Protein ABHD11 | 0.83 | 0.029 |
| Q13325 | IFIT5 | Interferon-induced protein with tetratricopeptide repeats 5 | 0.83 | 0.038 |
| O43447 | PPIH | Peptidyl-prolyl cis-trans isomerase H | 0.83 | 0.046 |
| Q8NEM2 | SHCBP1 | SHC SH2 domain-binding protein 1 | 0.83 | 0.022 |
| Q86X76 | NIT1 | Nitrilase homolog 1 | 0.83 | 0.045 |
| Q9Y3A3 | MOB4 | MOB-like protein phocein | 0.83 | 0.021 |
| G5E977 | NAPRT | Nicotinate phosphoribosyltransferase | 0.82 | 0.030 |
| P20591 | MX1 | Interferon-induced GTP-binding protein Mx1 | 0.82 | 0.027 |
| B9A018 | USP39 | U4/U6.U5 tri-snRNP-associated protein 2 | 0.82 | 0.006 |
| Q9UKX5 | ITGA11 | Integrin alpha-11 | 0.82 | 0.032 |
| Q9H2K0 | MTIF3 | Translation initiation factor IF-3, mitochondrial | 0.82 | 0.025 |
| C9JAZ1 | MTX2 | Metaxin-2 (Fragment) | 0.82 | 0.014 |
| Q93077 | HIST1H2AC | Histone H2A type 1-C | 0.82 | 0.015 |
| P33121 | ACSL1 | Long-chain-fatty-acid--CoA ligase 1 | 0.81 | 0.023 |
| D6RGG3 | COL12A1 | Collagen alpha-1(XII) chain | 0.81 | 0.001 |
| Q9NUM4 | TMEM106B | Transmembrane protein 106B | 0.81 | 0.000 |
| Q8IXI1 | RHOT2 | Mitochondrial Rho GTPase 2 | 0.81 | 0.011 |
| Q9NSY1 | BMP2K | BMP-2-inducible protein kinase | 0.81 | 0.024 |
| Q96TC7 | RMDN3 | Regulator of microtubule dynamics protein 3 | 0.81 | 0.019 |
| Q7L2E3 | DHX30 | Putative ATP-dependent RNA helicase DHX30 | 0.81 | 0.027 |
| G3V583 | FAM177A1 | Protein FAM177A1 (Fragment) | 0.81 | 0.014 |
| A0A0A0MTR7 | RNF213 | E3 ubiquitin-protein ligase RNF213 | 0.80 | 0.025 |
| Q9NPA0 | EMC7 | ER membrane protein complex subunit 7 | 0.80 | 0.013 |
| Q9BW92 | TARS2 | Threonine--tRNA ligase, mitochondrial | 0.80 | 0.008 |
| Q9BXW6 | OSBPL1A | Oxysterol-binding protein-related protein 1 | 0.80 | 0.033 |
| Q5T9L3 | WLS | Protein wntless homolog | 0.79 | 0.047 |
| Q96JH7 | VCPIP1 | Deubiquitinating protein VCIP135 | 0.79 | 0.019 |
| Q9NRG9 | AAAS | Aladin | 0.79 | 0.016 |
| Q92896 | GLG1 | Golgi apparatus protein 1 | 0.79 | 0.000 |
| Q8NBF2 | NHLRC2 | NHL repeat-containing protein 2 | 0.78 | 0.029 |
| P61962 | DCAF7 | DDB1- and CUL4-associated factor 7 | 0.78 | 0.025 |
| Q9Y6D9 | MAD1L1 | Mitotic spindle assembly checkpoint protein MAD1 | 0.78 | 0.017 |
| P02452 | COL1A1 | Collagen alpha-1(I) chain | 0.77 | 0.009 |
| Q04771 | ACVR1 | Activin receptor type-1 | 0.77 | 0.035 |
| Q14657 | LAGE3 | EKC/KEOPS complex subunit LAGE3 | 0.77 | 0.012 |
| Q99538 | LGMN | Legumain | 0.77 | 0.034 |
| P82675 | MRPS5 | 28S ribosomal protein S5, mitochondrial | 0.77 | 0.010 |
| P22681 | CBL | E3 ubiquitin-protein ligase CBL | 0.76 | 0.011 |
| Q4VC31 | CCDC58 | Coiled-coil domain-containing protein 58 | 0.75 | 0.046 |
| Q9BXI6 | TBC1D10A | TBC1 domain family member 10A | 0.75 | 0.046 |
| Q5JTZ9 | AARS2 | Alanine--tRNA ligase, mitochondrial | 0.75 | 0.027 |
| Q9UBU9 | NXF1 | Nuclear RNA export factor 1 | 0.74 | 0.021 |
| P62380 | TBPL1 | TATA box-binding protein-like protein 1 | 0.74 | 0.003 |
| Q9NXH9 | TRMT1 | tRNA (guanine(26)-N(2))-dimethyltransferase | 0.73 | 0.034 |
| Q96AX1 | VPS33A | Vacuolar protein sorting-associated protein 33A | 0.72 | 0.030 |
| A0A087X1I8 | ARMC9 | LisH domain-containing protein ARMC9 | 0.72 | 0.050 |
| Q4G0N4 | NADK2 | NAD kinase 2, mitochondrial | 0.70 | 0.044 |
| Q9NP92 | MRPS30 | 28S ribosomal protein S30, mitochondrial | 0.70 | 0.011 |
| Q9NSK0 | KLC4 | Kinesin light chain 4 | 0.70 | 0.032 |
| Q96PD2 | DCBLD2 | Discoidin, CUB and LCCL domain-containing protein 2 | 0.69 | 0.038 |
| E7EWV1 | PIGG | GPI ethanolamine phosphate transferase 2 | 0.69 | 0.044 |
| O43678 | NDUFA2 | NADH dehydrogenase [ubiquinone] 1 alpha subcomplex subunit 2 | 0.68 | 0.005 |
| Q9H425 | C1orf198 | Uncharacterized protein C1orf198 | 0.68 | 0.022 |
| F6SYF8 | DKK3 | Dickkopf-related protein 3 | 0.68 | 0.021 |
| P81605 | DCD | Dermcidin | 0.66 | 0.003 |
| Q9BQ95 | ECSIT | Evolutionarily conserved signaling intermediate in Toll pathway, mitochondrial | 0.64 | 0.042 |
| Q14012 | CAMK1 | Calcium/calmodulin-dependent protein kinase type 1 | 0.64 | 0.042 |
| P63272 | SUPT4H1 | Transcription elongation factor SPT4 | 0.63 | 0.021 |
| Q6NZY7 | CDC42EP5 | Cdc42 effector protein 5 | 0.63 | 0.013 |
| A0A0A0MR88 | FAM21C | WASH complex subunit FAM21C | 0.59 | 0.029 |
| B5MCP4 | LBH | Protein LBH | 0.57 | 0.045 |
| Q96II8 | LRCH3 | Leucine-rich repeat and calponin homology domain-containing protein 3 | 0.56 | 0.010 |
| P06702 | S100A9 | Protein S100-A9 | 0.56 | 0.007 |

**Figure S1** Direct cell counting of hPDLCs number after hypoxic treatment (n=3). Data are represented as mean ± SEM,; ^*^P < 0.05, ^**^P < 0.01 by two-tailed Student’s t test.
